# Club Cell Secretory Protein-16 (CC16) as a Prognostic Biomarker for COVID-19 and H1N1 Viral Infections

**DOI:** 10.3390/diagnostics14161720

**Published:** 2024-08-08

**Authors:** Shane Moore, Keerthana Gopichandran, Elizabeth Sevier, Siddhika Gamare, Sultan Almuntashiri, Gustavo Ramírez, Nora Regino, Luis Jiménez-Alvarez, Alfredo Cruz-Lagunas, Tatiana S. Rodriguez-Reyna, Joaquin Zuñiga, Caroline A. Owen, Xiaoyun Wang, Duo Zhang

**Affiliations:** 1 Department of Clinical and Administrative Pharmacy, College of Pharmacy, University of Georgia, Augusta, GA 30912, USA; 2 Department of Clinical Pharmacy, College of Pharmacy, University of Hail, Hail 55473, Saudi Arabia; 3 Laboratory of Immunobiology and Genetics, Instituto Nacional de Enfermedades Respiratorias Ismael Cosío Villegas, Tlalpan 4502, Mexico City 14080, Mexico; 4 Tecnologico de Monterrey, School of Medicine and Health Sciences, Mexico City 14380, Mexico; 5 Department of Immunology and Rheumatology, Instituto Nacional de Ciencias Médicas y Nutrición Salvador Zubirán, Tlalpan, Mexico City 14080, Mexico; 6 Division of Pulmonary and Critical Care Medicine, Brigham and Women’s Hospital, Harvard Medical School, Boston, MA 02115, USA; 7 Department of Medicine, Medical College of Georgia, Augusta University, Augusta, GA 30912, USA

**Keywords:** uteroglobin, influenza A virus, acute lung injury, acute respiratory distress syndrome, hospital stay

## Abstract

Severe Acute Respiratory Syndrome Coronavirus 2 (SARS-CoV-2) and H1N1 viruses are inflammatory lung pathogens that can lead to acute lung injury (ALI) and acute respiratory distress syndrome (ARDS). ALI/ARDS are still life-threatening diseases in critically ill patients with 30–40% mortality in the last decade. Currently, there are no laboratory tests for the early diagnosis or prognosis of ALI/ARDS. Club cell secretory protein (CC16) has been investigated as a potential biomarker of lung epithelial damage in various lung diseases. In this study, we evaluated whether plasma CC16 reflects the severity of COVID-19 and H1N1 infections. The plasma CC16 levels showed no significant differences between H1N1 and COVID-19 groups (*p* = 0.09). Among all subjects, CC16 levels were significantly higher in non-survivors than in survivors (*p* = 0.001). Upon the area under the receiver operating characteristic (AUROC) analysis, CC16 had an acceptable value to distinguish survivors and non-survivors (*p* = 0.002). In the COVID-19 group, plasma CC16 levels moderately correlated with the Acute Physiology and Chronic Health Evaluation II (APACHE II) score (r = 0.374, *p* = 0.003) and Sequential Organ Failure Assessment (SOFA) score (r = 0.474, *p* < 0.001). In the H1N1 group, a positive correlation was observed between the CC16 levels and hospital length of stay (r = 0.311, *p* = 0.022). Among all the patients, weak correlations between plasma CC16 levels with the SOFA score (r = 0.328, *p* < 0.001) and hospital length of stay (r = 0.310, *p* < 0.001) were observed. Thus, circulating CC16 might reflect the severity of COVID-19 and H1N1 infections.

## 1. Introduction

Severe Acute Respiratory Syndrome Coronavirus 2 (SARS-CoV-2) is a virus that caused the outburst of the COVID-19 pandemic [1]. Coronaviruses are enveloped single-stranded non-segmented RNA viruses belonging to the family *Coronaviridae*, specifically within the subfamily *Coronavirinae*. Coronaviruses are further classified into four genera, including alphacoronaviruses, betacoronaviruses, and deltacoronaviruses, based on phylogenetic analyses [2]. COVID-19 is the third documented spillover of an animal coronavirus to humans in only two decades, which resulted in a global pandemic and caused significant morbidity and mortality [2,3]. Several metabolic disorders, including obesity, diabetes, and kidney disease, have been associated with an increased severity of coronavirus diseases [4]. Cardiovascular conditions such as hypertension, congenital heart disease, and coronary artery disease not only predispose individuals to more severe illness, but can also exacerbate pre-existing conditions following recovery [5]. Age is also a significant risk factor, with older individuals being more likely to experience severe manifestations of COVID-19 [6]. The symptoms range from mild respiratory illness to severe pneumonia, acute lung injury (ALI), and its severe form, acute respiratory distress syndrome (ARDS) [7]. Once ARDS develops, patients usually have varying degrees of pulmonary artery vasoconstriction and may develop pulmonary hypertension [7]. Currently, although multiple COVID-19-associated biomarkers have been reported [8], lung-specific biomarkers, such as surfactant proteins, still need to be evaluated in these patients.

Influenza A Virus (IAV) is another infectious respiratory virus, which is known to have caused several pandemics [9]. It is a highly contagious single-stranded RNA virus; particularly, the H1N1 subtype has been a recurrent pathogen having seasonal outbreaks which cause significant respiratory illness [10]. The H1N1 purely avian virus pandemic in 1918 was responsible for an estimated 675,000 U.S. deaths and 50 to 100 million deaths worldwide [11]. The Centers for Disease Control and Prevention (CDC) estimated that seasonal IAV impacted an average of 12,000–52,000 deaths annually between 2010 and 2020 in the U.S. before the onset of the COVID-19 pandemic [11]. Influenza A, being a highly mutable virus, contributes to the annual variations in flu strains and the ongoing challenge in vaccines as it emerges at unpredictable intervals [12]. Most severe cases occur in noticeably young and elderly individuals [11]. The influenza virus targets the airway epithelial cells spanning the upper regions, such as the nasal passages, down to the lower regions of the respiratory tract reaching the alveoli [13]. The severity of the illness is dependent on how much the virus penetrates the lower respiratory tract [13]. Both SARS-CoV-2 and H1N1 viruses are inflammatory lung pathogens that cause severe lower respiratory tract inflammation and lung injury. These pathogens are associated with ALI/ARDS and cause a greater number of mortalities in infected patients [9,10]. Significantly, both coronavirus and influenza viruses are known to be airborne and can target the same respiratory tissues in humans, such as the nasal and bronchial epithelial cells [10]. The clinical manifestations of these viruses often resemble each other with symptoms, like cough, fever, and pneumonia, and are commonly associated with lower respiratory tract disease [14]. Furthermore, there is a circulation of seasonal endemic coronavirus infections with influenza virus infections [10]. Given their continued presence in the human population, there is a potential possibility for individuals to be co-infected with these respiratory viruses [10].

Mechanistically, SARS-CoV-2 and H1N1 cause ALI/ARDS by the release of pro-inflammatory cytokines, such as interleukin-1 (IL-1), IL-6, and Interferon-γ, which recruit neutrophils into the lungs [10]. The neutrophils are activated and release toxic mediators that damage the alveolar epithelium and endothelium and result in alveolar edema [15]. Alveolar edema prevents the gas exchange resulting in ALI/ARDS [7]. ALI/ARDS can develop gradually over a few days or can rapidly worsen. The clinical manifestations of ALI/ARDS are dyspnea, tachypnea, and hypoxemia. A chest X-ray showing bilateral pulmonary opacities, new or worsening respiratory symptoms, respiratory failure, or fluid overload are all diagnostic criteria [16]. ALI/ARDS can be life-threatening if not treated promptly. Currently, there are no laboratory tests for early diagnosis or prognosis [17].

Our team focuses on the investigation of circulating biomarkers for predicting the severity of ALI/ARDS, such as Matrix Metalloproteinase-3 (MMP-3) [18], Tissue Inhibitor of Metalloproteinase-1 (TIMP-1) [19], microvesicle-encapsulated microRNA-223 [17], and Club cell secretory protein (CC16) [20,21], which is also known as CC10 or uteroglobin. CC16 is a 10–16 kDa protein, primarily secreted by non-ciliated bronchial epithelial cells within the respiratory epithelium [22]. CC16 is easily detectable in the circulatory system under both normal and pathological conditions [22,23]. CC16 has been studied as a possible biomarker of lung epithelial damage in several kinds of diseases including ARDS, chronic obstructive pulmonary disease (COPD), pulmonary fibrosis, asthma, and sarcoidosis [22,23]. For example, it has been shown that circulating CC16 levels are increased in patients with ALI/ARDS [22,24]. While cigarette smoking can reduce the circulating CC16 levels [24], generally speaking, a transient increase in circulating CC16 is commonly seen due to the increased pulmonary vascular permeability caused by acute lung damage. In contrast, the decrease in circulating CC16 could be due to the suppression of *CC16* gene expression or the reduction in the club cells number in chronic environmental exposures [24].

Functionally, CC16 has an anti-inflammatory effect and protects against oxidative stress and apoptosis [25,26]. CC16 has a protective effect against the respiratory inflammatory response by modulating the activities of phospholipases A2, interferon-γ, tumor necrosis factor-α (TNF-α), and NF-κB [22,23,25,27,28]. Given its role in modulating inflammation and oxidative stress, CC16 may become integral in improving patient prognosis and tailoring interventions to combat COVID-19 and influenza viral infections.

Previously, our work demonstrated the utility of plasma CC16 in predicting 90-day mortality in two independent cohorts of patients with ARDS [20,21]. The objective of our current study is to determine whether plasma CC16 concentrations reflect the severity of COVID-19 and H1N1 infection.

## 2. Materials and Methods

### 2.1. Human Plasma Specimens and Ethics Approval

This is a retrospective analysis using unidentifiable human plasma specimens and determined as non-human subject research. Sixty plasma specimens from hospitalized patients with COVID-19 were collected between March and October 2020. Sixty-one plasma samples collected from patients diagnosed with H1N1 viral infection have been explained in detail previously [29]. Plasma samples from infected patients were collected at the emergency room from the Instituto Nacional de Enfermedades Respiratorias Ismael Cosío Villegas and from Instituto Nacional de Ciencias Médicas y Nutrición Salvador Zubirán, Mexico City. The detection of SARS-CoV-2 was performed by real-time polymerase chain reaction (RT-PCR) in swab samples, bronchial aspirates (BA), or bronchoalveolar lavage (BAL) specimens, as previously described [30]. H1N1 detection was first screened for influenza A virus infection using the Fuji dri-chem immuno AG cartridge FluAB kit (Fujifilm Corp, Tokyo, Japan) rapid influenza diagnostic test (RIDT) in fresh respiratory swab specimens. In positive cases, the further molecular characterization of the causative influenza A virus subtype was assessed by RT-PCR [30]. All influenza cases enrolled in the study were infected with the pandemic influenza A(H1N1) pdm09 virus. None of the participants had human immunodeficiency virus (HIV) infection.

Peripheral blood samples were obtained in the emergency department or within 48 h after patient admission to the hospital. Blood was obtained using standard phlebotomy techniques and ethylenediaminetetraacetic acid-anticoagulated collection tubes. Blood samples were layered over Ficoll in the laboratory and centrifuged at 400× *g* for 30 min at room temperature. The plasma layer was removed, centrifuged for 5 min at 350× *g* to remove residual peripheral blood mononuclear cells, and then was frozen in an ultra-low-temperature freezer until analysis. The severity of illness scores at admission, including the Sequential Organ Failure Assessment (SOFA), the Acute Physiology and Chronic Health Evaluation II (APACHE-II), and the partial pressure of oxygen/fraction of inspired oxygen ratio (PaO_2_/FiO_2_), were recorded.

The studies involving human participants were reviewed and approved by Institutional Review Boards of the Instituto Nacional de Ciencias Médicas y Nutrición Salvador Zubirán (INCMNSZ, approval number: 3349) and the Instituto Nacional de Enfermedades Respiratorias Ismael Cosío Villegas (INER, approval number: B28-16 and B09-20). The patients/participants provided their written informed consent to participate in this study. The study is also approved by the Augusta University Institutional Review Board (“Decoding the Molecular Mechanisms of Lung Diseases”, IRB number: 2070085, initial approval date 20 June 2023). All research procedures were in accordance with the ethical standards of the Augusta University Institutional Review Board and with the amended Helsinki Declaration of 1975.

### 2.2. Enzyme-Linked Immunosorbent Assay (ELISA)

The ELISA was performed using a CC16 ELISA kit designed and validated for human use (R&D Systems, Catalog # DY4218, Minneapolis, MN, USA) according to the manufacturer’s instructions, as previously described [28]. The ELISA range is 31.2–2000 pg/mL. Plasma samples were stored at −80 °C before the study, and freeze–thaw cycles were less than five. No further freeze or thaw steps were taken during the study. Human plasma samples were diluted 40 times using reagent diluent (DY995, R&D Systems). Diluted plasma samples were measured in duplicates to determine the CC16 concentration in 100 µL human plasma. Plate incubation and absorbance measurement at a wavelength of 450 nm was conducted using the SpectraMax M5 Microplate Reader (Molecular Devices LLC., Sunnyvale, CA, USA).

### 2.3. Statistical Analysis

Data were analyzed using IBM SPSS Statistics Version 27.0 and figures were developed with GraphPad Prism Vesion 9.0.0. Statistical significance was set at an alpha of 0.05. AUROC analysis was performed using GraphPad Prism Vesion 9.0.0. Patient demographic data were evaluated. Pearson correlation was used to evaluate relationships between CC16 and prognostic parameters. The Shapiro–Wilk test was used for the test of normality. For normal distribution data, the mean and standard deviation were calculated, whereas median and interquartile ranges were reported for nonnormal distribution data. Student’s t-tests or Mann–Whitney U tests were performed to calculate the *p* value.

## 3. Results

### 3.1. Study Population

The demographic information, baseline characteristics, and outcomes of COVID-19 and H1N1 patients are shown in Table 1. There were no significant differences were seen in age and gender between the groups (*p* > 0.05). Significant differences are found between COVID-19 and H1N1 patients in the ratio of PaO_2_/FiO_2_, APACHE II score, SOFA score, and hospital length of stay toward more unfavorable outcomes in H1N1 patients (all *p* < 0.001).

### 3.2. CC16 Levels in COVID-19 and H1N1 Patients

**Figure 1:**
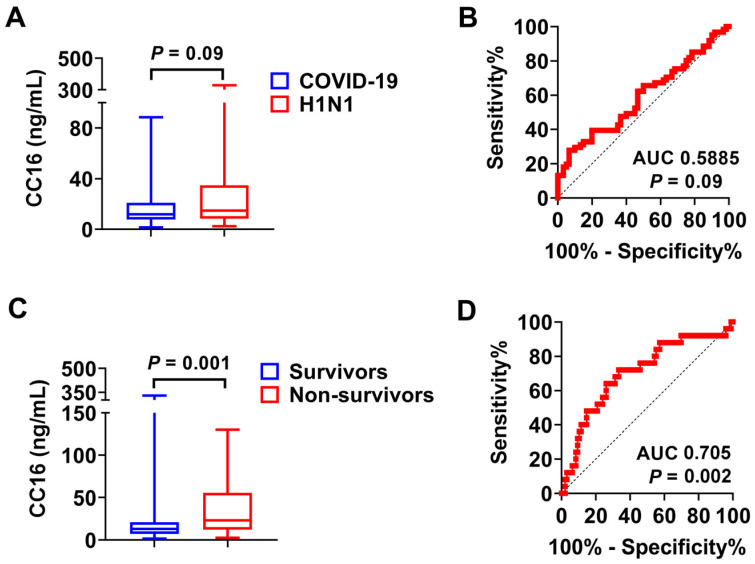
Comparison of plasma CC16 in various groups. (**A**) COVID-19 (*n* = 60) versus H1N1 (*n* = 61). (**B**) ROC curve to distinguish between COVID-19 and H1N1 based on CC16 level. (**C**) Survivors (*n* = 96) vs. non-survivors (*n* = 25). (**D**) ROC curve to distinguish between survivors and non-survivors based on CC16 level.

We measured the concentrations of plasma CC16 from COVID-19 patients (*n* = 60) versus H1N1 patients (*n* = 61). The median levels of CC16 were 12.04 ng/mL and 14.92 ng/mL in the COVID-19 group and H1N1 group, respectively. There was no significant difference between these two groups (Figure 1A, *p* = 0.09, COVID-19 vs. H1N1). Upon the AUROC analysis of CC16 to distinguish the differences between COVID-19 and H1N1 groups, CC16 had a poor AUC value of 0.589 (Figure 1B, 95% confidence interval [CI]: 0.487 to 0.690; *p =* 0.09). Previously, we reported that the median level of plasma CC16 in the healthy control (*n* = 20) was 8.396 ng/mL [21]. In this case, plasma CC16 levels were significantly higher in the COVID-19 (*p* = 0.0147, COVID-19 vs. healthy control) and H1N1 groups (*p* = 0.0004, H1N1 vs. healthy control) than those in the healthy control, respectively.

In contrast, the plasma levels of CC16 were significantly higher in non-survivors when compared with the levels in survivors. The median level of CC16 in the survivor group (*n* = 96) was 13.15 ng/mL. In contrast, the median level of CC16 from non-survivors (*n* = 25) was 23.48 ng/mL (Figure 1C, *p* = 0.001, survivors vs. non-survivors). Upon the AUROC analysis of CC16 to distinguish the differences between survivors and non-survivors, CC16 had an acceptable AUC value of 0.705 (Figure 1D, 95% CI: 0.585 to 0.824; *p =* 0.002).

### 3.3. The Relation of Plasma CC16 Level to the Severity of COVID-19

**Figure 2:**
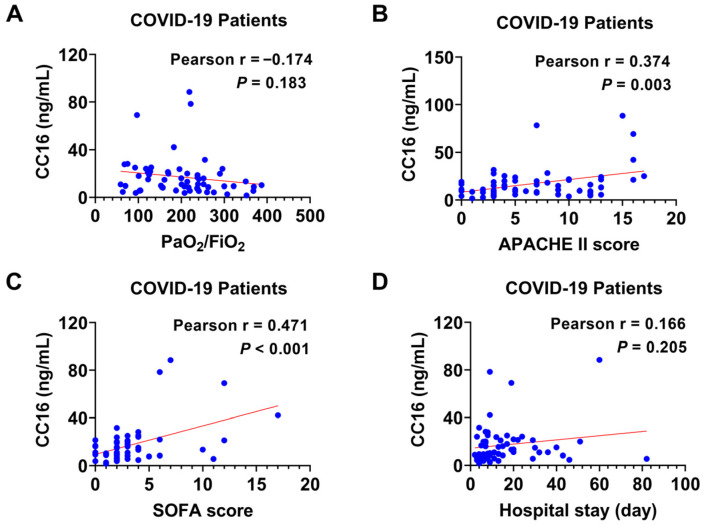
Correlation analyses between plasma CC16 and the prognostic parameters in COVID-19 patients. Pearson correlation was used to evaluate relationships between (**A**) CC16 and PaO_2_/FIO_2_ (*n* = 60), (**B**) CC16 and APACHE III score (*n* = 60), (**C**) CC16 and SOFA score (*n* = 60), and (**D**) CC16 and hospital length of stay (*n* = 60).

We further analyzed the correlations of plasma CC16 with the PaO_2_/FiO_2_ ratio (Figure 2A, r = −0.174, 95% CI: −0.410 to 0.083, *p =* 0.183), APACHE II score (Figure 2B, r = 0.374, 95% CI: 0.132 to 0.573, *p =* 0.003), SOFA score (Figure 2C, r = 0.471, 95% CI: 0.247 to 0.648, *p* < 0.001) and hospital length of stay (Figure 2D, r = 0.310, 95% CI: −0.092 to 0.403, *p =* 0.205) in COVID-19 patients.

### 3.4. The Relation of Plasma CC16 Level to the Severity of H1N1 Infection

**Figure 3:**
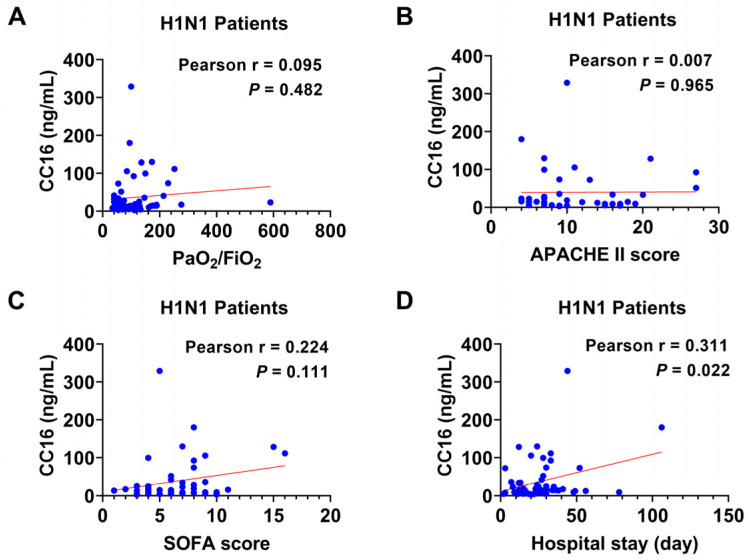
Correlation analyses between plasma CC16 and the prognostic parameters in H1N1 patients. Pearson correlation was used to evaluate relationships between (**A**) CC16 and PaO_2_/FIO_2_ (*n* = 57), (**B**) CC16 and APACHE III score (*n* = 43), (**C**) CC16 and SOFA score (*n* = 52), and (**D**) CC16 and hospital length of stay (*n* = 54).

In the H1N1 group, there were no significant correlations between CC16 levels and the PaO_2_/FiO_2_ ratio (Figure 3A, r = 0.095, 95% CI: −0.170 to 0.347, *p =* 0.482), APACHE II score (Figure 3B, r = 0.007, 95% CI: −0.294 to 0.307, *p =* 0.965), or SOFA score (Figure 3C, r = 0.224, 95% CI: −0.052 to 0.468, *p =* 0.111). Only a weak positive correlation was observed between plasma CC16 levels and the hospital length of stay in infected patients (Figure 3D, r = 0.311, 95% CI: 0.047 to 0.534, *p* = 0.022).

### 3.5. The Correlation between Plasma CC16 and the Disease Severity in Combined Groups

**Figure 4:**
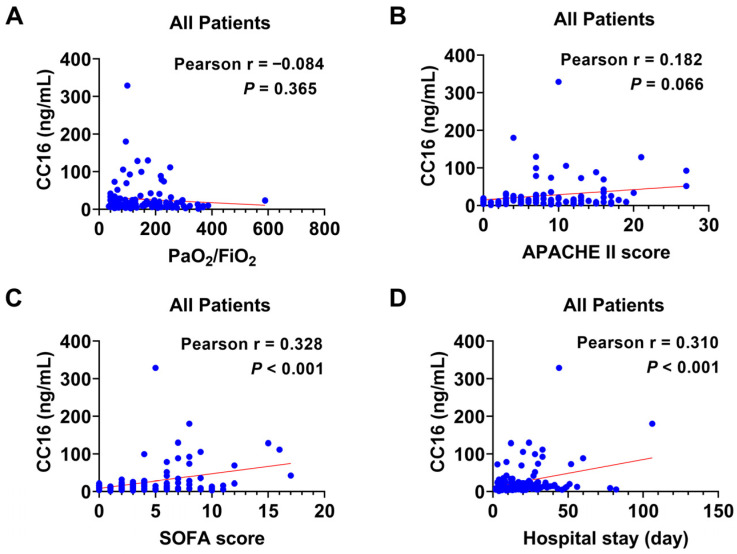
Correlation analyses between plasma CC16 and the prognostic parameters in infected patients from COVID-19 and H1N1 groups. Pearson correlation was used to evaluate relationships between (**A**) CC16 and PaO_2_/FIO_2_ (*n* = 117), (**B**) CC16 and APACHE III score (*n* = 103), (**C**) CC16 and SOFA score (*n* = 112), and (**D**) CC16 and hospital length of stay (*n* = 114).

Next, we combined both groups and conducted the same correlation analysis. Among all the patients in the study, there were no significant correlations between the CC16 level and the PaO_2_/FiO_2_ ratio (Figure 4A, r = −0.084, 95% CI: −0.262 to 0.099, *p =* 0.365) or APACHE II score (Figure 4B, r = 0.182, 95% CI: −0.012 to 0.363, *p =* 0.066). In contrast, we found that plasma CC16 levels have significant positive correlations with the SOFA score (Figure 4C, r = 0.328, 95% CI: 0.152 to 0.484, *p* < 0.001) and hospital length of stay (Figure 4D, r = 0.310, 95% CI: 0.134 to 0.467, *p* < 0.001). To be noted, both correlations were weak considering that the r values were less than 0.33.

## 4. Discussion

ARDS is a common, lethal, and heterogeneous syndrome with no approved pharmacotherapeutic treatments or laboratory-based diagnostic or prognostic tests. For years, researchers have focused on the identification of biomarkers for the diagnosis and prognosis of ARDS. For instance, surfactant protein D (SP-D), as a marker of lung epithelial damage, may serve as a potential biomarker for the diagnosis of ARDS in several studies [31,32]. Elevated pro-inflammatory cytokines in the circulation are also associated with the severity of lung injury, such as IL-6 and TNF-α [33].

Given that CC16 is a protein highly expressed in club cells, it is recognized as a useful indicator for club cell dysfunction among the reported biomarkers [34]. Rohmann et al. showed that serum CC16 was significantly increased in severe SARS-CoV-2 and severe non-pulmonary sepsis than in healthy controls, but no distinct differences between direct and indirect lung infections [35]. In severe SARS-CoV-2 infections, circulating CC16 levels were positively correlated to disease duration and negatively to the platelet count, reinforcing the high potential of CC16 as a biomarker for epithelial cell damage and air–blood barrier leakage in pulmonary or non-pulmonary infectious diseases [35]. In addition, a research group aimed to determine the predictive value of CC16 on the incidence of ARDS or death in patients with COVID-19. They found that the initial CC16 level on day 1 was positively correlated with fatal outcomes, which supports the usefulness of CC16 as a predictor of mortality in patients with COVID-19 [36].

The disease states that participants in this study had that then progressed to ARDS were either COVID-19 or influenza, H1N1 more specifically. In both disease states, the mechanism of action involves pulmonary infiltration when these viruses attack the epithelial cells, leading to an inflammatory response [37]. This inflammatory response is triggered by infected epithelial cells creating cytokines, which then lead to the production of neutrophils and macrophages. These neutrophils and macrophages can create reactive oxygen species as well as nitric oxide which can cause severe damage to the epithelial barrier in the lungs. Furthermore, macrophages, in some cases, can directly kill epithelial cells through apoptosis, especially in response to the infection of influenza-like viruses [37]. As a compensatory mechanism, the production and secretion of CC16 protein could increase. This protein helps the respiratory tract to inhibit inflammation, as well as reducing the reactive oxygen species, which might explain the increased release of CC16 during acute lung damage [27].

The results in this study were similar to what was described in a previous analysis study of CC16 in ARDS [21]. Chase et al. reported that elevated initial CC16 levels in ARDS correlated with worse outcomes with regard to 90-day mortality by a significant amount [21], and this finding was further validated in an independent cohort [20]. A different observational study by Lin et al. reported that an elevated CC16 concentration correlated with worse outcomes in regard to the ICU length of stay, mortality, and a correlation between the severity of ARDS and CC16 levels based on the PaO_2_/FiO_2_ ratio [38]. In this study, we found similar correlations in the elevation of CC16 in non-survivors. Also, some significant correlations between the severity of diseases and CC16 levels were observed. However, these preliminary findings require further validation using a larger sample size considering that the correlations were not strong.

This pilot study had some significant limitations. First, the study design was retroactive which prevented any inferential conclusions from being drawn. Second, this study had a small sample size, as stated above, which could limit the generalizability of these results to a larger population. The third limitation involves baseline differences (e.g., APACHE II and SOFA) between the COVID-19 and H1N1 groups, indicating that they are not of comparable severity. Therefore, it could be misleading when they were analyzed together. In addition, several confounders were not considered in the study. For example, there were no records indicating whether the patients experienced bacterial co-infection during hospitalization. Furthermore, the lack of analysis of samples from healthy donors makes it difficult to interpret the CC16 concentration in clinical settings without the normal CC16 range. Lastly, it is known that the combination of various biomarkers is usually more accurate than a single one [39]. In the future study, we aim to combine multiple biomarkers to improve diagnostic performance. Despite these limitations, circulating CC16 was still significantly associated with mortality and showed some potential for being used as a prognostic biomarker in COVID-19 and H1N1 patients.

In conclusion, our study determined the potential application of CC16 as a prognostic biomarker for COVID-19 or H1N1 patients. Particularly, circulating CC16 was more increased in non-survivors than in survivors. Its levels were also associated with the severity of COVID-19 as reflected by the APACHE II score and SOFA score. In the H1N1 group, CC16 levels correlated with the length of hospital stay. When analyzing all the patients together, CC16 plasma levels were associated with the SOFA score and hospital length of stay, but not the PaO_2_/FiO_2_ ratio or APACHE II score. However, the strength of the most significant correlations was weak, such as CC16 levels with the SOFA score (Figure 4C) and length of stay (Figure 4D), which might be caused by the small sample size. Therefore, we aim to further this investigation with a large sample size determined by power analysis.

## Figures and Tables

**Table 1:** Demographic characteristics of COVID-19 and H1N1 patients.

	All (*n* = 121)	COVID-19 (*n* = 60)	H1N1 (*n* = 61)	*p* Value ^1^
Age in years	49.09 (12.15)	50.30 (12.96)	47.90 (11.27)	0.28
Female gender *n* (%)	60 (49.6)	30 (50)	30 (49.2)	0.92
PaO_2_/FiO_2_	130 (79–219.5)	207.5 (124.25–252)	92 (60–141.5) ^2^	<0.001
>300 *n* (%)	8 (6.84)	7 (11.67)	1 (1.75)	0.10
201–300 *n* (%)	29 (24.79)	25 (41.67)	4 (7.02)	<0.001
101–200 *n* (%)	38 (32.48)	20 (33.33)	18 (31.58)	0.98
≤100 *n* (%)	42 (35.90)	8 (13.33)	34 (59.65)	<0.001
APACHE II	7 (4–12)	5 (3–10.75)	10 (7–16) ^3^	<0.001
SOFA	4 (2–7)	2 (2–4)	6 (4–8) ^4^	<0.001
Hospital stay in days	17 (8–29.25)	9 (6–20)	25.5 (15–33) ^5^	<0.001
Survivors *n* (%)	96 (79.34)	47 (78.33)	49 (80.33)	0.96

^1^ Statistical test showing the comparison between COVID-19 and H1N1 groups. ^2^ *n* = 57. Four patients’ PaO_2_/FiO_2_ in H1N1 group were unavailable. ^3^ *n* = 43. Eighteen patients’ APACHE II in the H1N1 group were unavailable. ^4^ *n* = 52. Nine patients’ SOFA in the H1N1 group were unavailable. ^5^ *n* = 54. Seven patients’ hospital stay in the H1N1 group were unavailable. Definition of abbreviations: PaO_2_: Partial pressure of oxygen (PaO_2_); FiO_2_: Fraction of inspired oxygen; APACHE II: Acute Physiology and Chronic Health Evaluation II; SOFA: Sequential Organ Failure Assessment score. Data are presented as median (interquartile range) for data that were not normally distributed or mean ± SD for data that were normally distributed.

## Data Availability

Data will be made available on request from the corresponding author, D.Z.
